# A NET-associated immunothrombotic phenotype is associated with coronary no-reflow and myocardial injury after primary PCI in ST-segment elevation myocardial infarction: a real-world translational cohort

**DOI:** 10.3389/fimmu.2026.1926100

**Published:** 2026-09-29

**Authors:** Zhigang Sun, Zhidong Zhang, Gang Qiao, Fangyuan Jia

**Affiliations:** Department of Aortic Surgery, Central China Subcenter of National Center for Cardiovascular Diseases, Henan Cardiovascular Disease Center, Fuwai Central-China Cardiovascular Hospital, Central China Fuwai Hospital of Zhengzhou University, Zhengzhou, China

**Keywords:** citrullinated histone H3, immunothrombosis, MPO-DNA, myocardial injury, neutrophil extracellular traps, no-reflow, primary percutaneous coronary intervention, ST-segment elevation myocardial infarction

## Abstract

**Background:**

Coronary no-reflow after primary percutaneous coronary intervention (PCI) reflects failure of myocardial reperfusion despite treatment of the culprit epicardial lesion. Neutrophil extracellular traps (NETs) and immunothrombosis provide a plausible link between sterile inflammation, thrombus biology, and microvascular obstruction. We evaluated whether a clinically measurable NET-associated immunothrombotic phenotype (NET-IP) was associated with no-reflow and myocardial injury after primary PCI.

**Methods:**

This single-center translational cohort included 742 patients with ST-segment elevation myocardial infarction (STEMI) undergoing primary PCI. NET-IP was calculated in 725 patients from five admission or pre-PCI variables—neutrophil count, D-dimer, fibrinogen, high-sensitivity C-reactive protein, and platelet-neutrophil product—by averaging log-transformed component z scores when at least four components were available, followed by standardization of the composite score. The primary endpoint was angiographic no-reflow, defined as final thrombolysis in myocardial infarction flow grade < 3 in the absence of mechanical epicardial obstruction. A case-enriched nested enzyme-linked immunosorbent assay (ELISA) biomarker subcohort was used to measure MPO-DNA, citrullinated histone H3 (H3Cit), and double-stranded DNA.

**Results:**

Angiographic no-reflow occurred in 110 of 742 patients. NET-IP was calculable in 725 patients, and the fully adjusted complete-case models included 700 patients. Patients with no-reflow had higher admission NET-IP scores than those with normal reflow (0.73 [0.24, 1.25] vs. -0.06 [-0.77, 0.54]; *P* < 0.001). In the fully adjusted model, NET-IP remained associated with angiographic no-reflow (OR per SD, 1.96; 95% CI, 1.46–2.62; *P* < 0.001) and tissue-level reperfusion failure (OR per SD, 1.64; 95% CI, 1.32–2.02; *P* < 0.001). In the case-enriched ELISA subcohort, adjusted associations with no-reflow were observed for MPO-DNA (OR per SD log_2_, 1.88; 95% CI, 1.26–2.81; *P* = 0.002) and H3Cit (OR per SD log_2_, 2.01; 95% CI, 1.33–3.02; *P* < 0.001), whereas double-stranded DNA was weaker after adjustment (*P* = 0.074). NET-IP was also associated with higher peak high-sensitivity cardiac troponin I, higher peak creatine kinase-MB, lower discharge left ventricular ejection fraction (LVEF), large myocardial injury, and discharge LVEF < 50%.

**Conclusion:**

A NET-IP was linked to impaired coronary reperfusion and myocardial injury after primary PCI in STEMI. The findings support NET-associated immunothrombosis as a clinically measurable immune phenotype but do not establish direct NET burden, causal mediation, or immediate clinical treatment guidance.

## 1 Introduction

Primary percutaneous coronary intervention (PCI) restores epicardial patency in most patients with acute myocardial infarction (AMI), yet restoration of Thrombolysis in Myocardial Infarction (TIMI) 3 flow does not always translate into adequate tissue-level reperfusion. Coronary no-reflow represents this gap between opening the infarct-related artery and restoring microvascular blood flow, and it has been associated with larger infarction, adverse remodeling, heart failure, and mortality (1, 2). The concept is clinically important because it points to a pathobiology that is not solved by stent implantation alone.

No-reflow is not a single lesion. Distal thromboembolization, ischemic microvascular damage, endothelial swelling, vasoconstriction, platelet-leukocyte plugging, and reperfusion-related inflammation may converge in the same patient (3, 4). Angiographic markers such as final TIMI flow, myocardial blush grade (MBG), and corrected TIMI frame count (CTFC) remain practical tools for evaluating this process at the catheterization-laboratory level (5, 6). Cardiac magnetic resonance studies have also shown that microvascular obstruction provides prognostic information beyond infarct size in ST-segment elevation myocardial infarction (STEMI) cohorts (7).

The early immune response to AMI is necessary for debris clearance and tissue repair, but an excessive or poorly resolved innate immune response can amplify ischemia-reperfusion injury (8, 9). Neutrophils arrive early in the injured myocardium and can contribute through proteases, oxidants, endothelial interactions, and extracellular vesicles; at the same time, neutrophil-derived signals participate in subsequent repair programs (10–12). This dual biology is one reason why broad anti-inflammatory approaches in AMI require careful patient selection rather than indiscriminate immune suppression (13).

Neutrophil extracellular traps (NETs) add a thromboinflammatory layer to this problem. First described as chromatin-based antimicrobial structures, NETs contain DNA, histones, myeloperoxidase, neutrophil elastase, and other granular proteins (14). In the vasculature, NETs can serve as scaffolds for platelet binding and fibrin deposition and can connect innate immune activation with coagulation, a process often framed as immunothrombosis (15, 16). NET components can also interact with fibrin architecture and fibrinolysis, potentially stabilizing thrombi in a way that is relevant to microvascular plugging (17, 18).

Several observations support a role for NETs in AMI. NET burden in culprit coronary thrombi has been linked to ST-segment resolution and infarct size in ST-elevation acute coronary syndrome (19). Circulating NET-related components, including double-stranded DNA (dsDNA) and citrullinated histone H3 (H3Cit)-related signals, have been associated with infarct size, ventricular dysfunction, remodeling, and clinical outcomes after STEMI (20–22). Experimental ischemia-reperfusion studies further suggest that NET-mediated microthrombosis may contribute to myocardial no-reflow and that DNase-based strategies can attenuate this process in preclinical models (23).

Despite this evidence, a practical clinical problem remains. Direct NET assays are not routinely available in AMI care, while commonly used inflammatory indices such as neutrophil-to-lymphocyte ratio (NLR) or systemic immune-inflammation index (SII) are biologically nonspecific and do not explicitly integrate coagulation and platelet-neutrophil interaction (24, 25). A phenotype that combines neutrophil activation, fibrin turnover, fibrinogen-rich acute-phase response, systemic inflammation, and platelet-neutrophil interaction may better approximate the immunothrombotic state that precedes or accompanies reperfusion failure.

We therefore evaluated a defined NET-associated immunothrombotic phenotype score (NET-IP) in a real-world STEMI cohort undergoing primary PCI. The primary objective was to determine whether NET-IP was associated with angiographic no-reflow after adjustment for conventional clinical and angiographic predictors. Secondary objectives were to evaluate tissue-level reperfusion failure, myocardial injury, internal robustness, and biological anchoring against measured NET-associated markers in a case-enriched nested enzyme-linked immunosorbent assay (ELISA) biomarker subcohort.

## 2 Materials and methods

### 2.1 Study design and population

This was a single-center real-world translational cohort study conducted at Central China Fuwai Hospital of Zhengzhou University. Adult patients with STEMI undergoing primary PCI were included. Myocardial infarction was defined according to contemporary universal MI criteria (26). The cohort was assembled through clinical collaboration between the Aortic Surgery author team and the Interventional Cardiology/catheterization-laboratory service. The two independent angiographic readers were from the main author team and performed blinded image review. The study used routinely collected clinical, laboratory, angiographic, procedural, echocardiographic, and biomarker data. Biomarker assays used residual clinical samples collected during routine care; no additional research blood draw was performed. The same ethics approval (No. fwhzz02511041) covered the research use and biomarker analysis of these residual clinical samples. The protocol was reviewed and approved by the Ethics Committee of Central China Fuwai Hospital of Zhengzhou University, with a waiver of written informed consent for the retrospective use of de-identified clinical data.

The main cohort consisted of 742 eligible patients with analyzable angiographic outcome data. NET-IP was calculable in 725 patients and unavailable in 17 patients. A case-enriched nested ELISA biomarker subcohort comprised 228 patients with available residual clinical samples (150 with normal reflow and 78 with no-reflow). The subcohort was enriched for no-reflow cases to support biomarker validation and was not created by individual or frequency matching (**Supplementary Tables S4**-**S5**).

### 2.2 Exposure definition

The primary exposure was the NET-IP score. The five components were selected to capture complementary, clinically measurable domains of immunothrombosis: neutrophil abundance, fibrin turnover, fibrinogen-rich acute-phase response, systemic inflammation, and platelet-neutrophil interaction. Neutrophil count, D-dimer, fibrinogen, high-sensitivity C-reactive protein (hsCRP), and platelet-neutrophil product were log-transformed and individually standardized; the available component z scores were averaged when at least four of five components were present, and the resulting composite was standardized for analysis. The high NET-IP phenotype was defined as the upper tertile of the NET-IP distribution (**Supplementary Table S1**).

NLR and SII were analyzed as comparator inflammatory indices in sensitivity analyses. SII was calculated as platelet count × neutrophil count/lymphocyte count. Neither comparator was used to define the primary NET-IP exposure.

### 2.3 Angiographic and reperfusion endpoints

The primary endpoint was angiographic no-reflow, defined as final Thrombolysis in Myocardial Infarction (TIMI) flow grade < 3 after PCI in the absence of residual mechanical epicardial obstruction. Mechanical causes of impaired epicardial flow, including flow-limiting dissection, severe residual stenosis, persistent severe spasm, or acute vessel closure, were excluded by blinded angiographic adjudication. No patient in the analysis dataset had residual stenosis > 30% after PCI (**Table 1**).

**Table 1:** Angiographic predictors, procedural features, and reperfusion outcomes.

Characteristic	Overall (*n* = 742)	Normal reflow (*n* = 632)	No-reflow (*n* = 110)	*P* value
Culprit vessel	—	—	—	0.345
LAD	341 (46.0%)	284 (44.9%)	57 (51.8%)	
LCX	134 (18.1%)	120 (19.0%)	14 (12.7%)	
Left main	2 (0.3%)	2 (0.3%)	0 (0.0%)	
RCA	265 (35.7%)	226 (35.8%)	39 (35.5%)	
LAD or left main culprit	343 (46.2%)	286 (45.3%)	57 (51.8%)	0.202
Pre-PCI TIMI flow grade	—	—	—	< 0.001
0	262 (35.3%)	195 (30.9%)	67 (60.9%)	
1	229 (30.9%)	196 (31.0%)	33 (30.0%)	
2	145 (19.5%)	139 (22.0%)	6 (5.5%)	
3	106 (14.3%)	102 (16.1%)	4 (3.6%)	
Pre-PCI TIMI 0–1	491 (66.2%)	391 (61.9%)	100 (90.9%)	< 0.001
Pre-PCI thrombus grade	—	—	—	—
0	24 (3.2%)	23 (3.6%)	1 (0.9%)	
1	84 (11.3%)	81 (12.8%)	3 (2.7%)	
2	177 (23.9%)	158 (25.0%)	19 (17.3%)	
3	237 (31.9%)	213 (33.7%)	24 (21.8%)	
4	146 (19.7%)	108 (17.1%)	38 (34.5%)	
5	74 (10.0%)	49 (7.8%)	25 (22.7%)	
High thrombus burden	220 (29.6%)	157 (24.8%)	63 (57.3%)	< 0.001
Final TIMI flow grade	—	—	—	< 0.001
0	4 (0.5%)	0 (0.0%)	4 (3.6%)	
1	15 (2.0%)	0 (0.0%)	15 (13.6%)	
2	91 (12.3%)	0 (0.0%)	91 (82.7%)	
3	632 (85.2%)	632 (100.0%)	0 (0.0%)	
Myocardial blush grade	—	—	—	< 0.001
0	25 (3.4%)	0 (0.0%)	25 (22.7%)	
1	81 (10.9%)	17 (2.7%)	64 (58.2%)	
2	236 (31.8%)	215 (34.0%)	21 (19.1%)	
3	400 (53.9%)	400 (63.3%)	0 (0.0%)	
Corrected TIMI frame count	22.8 [18.9, 27.8]	21.6 [18.2, 24.9]	40.7 [35.5, 46.3]	< 0.001
Lesion length, mm	25.5 [20.3, 30.2]	25.5 [20.4, 30.3]	25.5 [19.7, 29.8]	0.772
Residual stenosis, %	9.6 [6.6, 12.8]	9.4 [6.5, 12.4]	12.3 [7.5, 14.5]	< 0.001
Multivessel disease	367 (49.5%)	304 (48.1%)	63 (57.3%)	0.076
Direct stenting	334 (45.0%)	291 (46.0%)	43 (39.1%)	0.176
Thrombus aspiration	251 (33.8%)	205 (32.4%)	46 (41.8%)	0.055
GP IIb/IIIa inhibitor use	325 (43.8%)	269 (42.6%)	56 (50.9%)	0.103
Intracoronary vasodilator use	114 (15.4%)	72 (11.4%)	42 (38.2%)	< 0.001
Procedural distal embolization	49 (6.6%)	38 (6.0%)	11 (10.0%)	0.120

Values are median [IQR] or n (%). TIMI denotes Thrombolysis in Myocardial Infarction; MBG, myocardial blush grade; CTFC, corrected TIMI frame count. Final TIMI flow, MBG, and CTFC are reported as reperfusion outcomes. Mechanical obstruction was excluded by blinded angiographic adjudication; residual stenosis > 30% was not present in the analysis dataset.

The key secondary reperfusion endpoint was tissue-level reperfusion failure, defined as final TIMI flow < 3 or final TIMI 3 with MBG 0–1 or adjudicated CTFC > 27. TIMI flow, MBG, and CTFC were assessed using established angiographic methods (5, 6). Interobserver agreement for no-reflow classification, MBG, and CTFC was summarized in **Supplementary Table S14**.

### 2.4 Myocardial injury outcomes

Myocardial injury outcomes included peak high-sensitivity cardiac troponin I (hs-cTnI), peak creatine kinase-MB (CK-MB), discharge left ventricular ejection fraction (LVEF), large myocardial injury, and discharge LVEF < 50%. Continuous hs-cTnI and CK-MB outcomes were log-transformed before regression modeling, and effect estimates were back-transformed to percent differences per SD increase in NET-IP. Large myocardial injury was defined as the top quartile of the peak myocardial injury marker in the analysis cohort (**Supplementary Table S1**).

### 2.5 Nested ELISA biomarker validation

MPO-DNA, H3Cit, and dsDNA were measured in the case-enriched nested ELISA biomarker subcohort. Sample type, sampling time relative to PCI, blood draw-to-centrifugation time, freeze-thaw count, ELISA plate, and batch were recorded. Duplicate and internal-control coefficient-of-variation quality control was summarized in **Supplementary Figure S2**. Exact sampling timing and pre-analytical sensitivity analyses are reported in **Supplementary Tables S6** and **S10**.

Circulating MPO-DNA complexes were quantified using a modified capture ELISA. High-binding 96-well plates were coated overnight at 4 °C with mouse anti-human MPO monoclonal antibody (clone 4A4; Bio-Rad; Cat. No. 0400-0002; 5 μg/ml). After blocking with 1% BSA in PBS, samples diluted 1:2 were added in duplicate and incubated for 2 h at room temperature. Bound MPO-DNA complexes were detected using the peroxidase-conjugated anti-DNA monoclonal antibody from the Cell Death Detection ELISA PLUS system (Roche Diagnostics/Sigma-Aldrich; Cat. No. 11774425001), followed by TMB development. Absorbance was measured at 450 nm with reference correction at 620 nm. Concentrations were expressed as arbitrary units per milliliter using a two-fold serially diluted internal NET calibrator and a four-parameter logistic calibration curve.

H3Cit was quantified using the Citrullinated Histone H3 (Clone 11D3) ELISA Kit (Cayman Chemical; Item No. 501620). Samples were diluted 1:5 in assay buffer and analyzed in duplicate. Standards or samples were incubated for 2h at room temperature, followed by a 1h incubation with the HRP-conjugated detection antibody. After TMB development for 30 min in the dark, absorbance was measured at 450 nm. Concentrations were determined using an 8-point standard curve and four-parameter logistic regression and corrected for the dilution factor.

Circulating dsDNA was quantified using the Quant-iT PicoGreen dsDNA Assay Kit (Invitrogen/Thermo Fisher Scientific; Cat. No. P11496). Samples were diluted 1:5 in TE buffer and analyzed in duplicate after addition of PicoGreen working reagent. Plates were incubated for 5 min at room temperature protected from light, and fluorescence was measured at approximately 485-nm excitation and 525-nm emission. Concentrations were calculated from a lambda-dsDNA calibration curve using blank-corrected linear regression.

### 2.6 Statistical analysis

Continuous variables are presented as median [IQR] and categorical variables as *n* (%). Between-group comparisons used Wilcoxon rank-sum, chi-square, or Fisher exact tests as appropriate. Global comparisons across NET-IP tertiles used Pearson chi-square tests for categorical variables and Kruskal–Wallis tests for continuous variables. Benjamini–Hochberg false-discovery-rate adjustment was applied to families of exploratory secondary comparisons. Missingness was summarized separately for main-cohort analytic variables and for NET-associated markers that were not assayed outside the nested ELISA subcohort (**Supplementary Table S2**).

Logistic regression estimated the association between NET-IP and angiographic no-reflow or tissue-level reperfusion failure. Model 1 was unadjusted. Model 2 adjusted for age, sex, diabetes, and symptom-to-balloon time. Model 3 additionally adjusted for Killip class ≥ 3, culprit left anterior descending artery (LAD) or left main, pre-PCI TIMI flow 0–1, high thrombus burden, admission glucose, and estimated glomerular filtration rate (eGFR). Restricted cubic splines were used for graphical dose-response assessment. Internal bootstrap validation was used to estimate optimism-corrected discrimination and the bootstrap distribution of the NET-IP coefficient. Multiple imputation by chained equations was performed as a sensitivity analysis for missing covariates among patients with a calculable NET-IP score; NET-IP itself was not imputed (27). Because this was a retrospective consecutive cohort, no formal sample-size calculation was performed; all eligible patients meeting the study criteria were included. The fully adjusted primary model contained 103 no-reflow events for 11 modeled coefficients excluding the intercept.

ELISA marker models were fitted per SD increase in log_2_-transformed marker levels. Clinical/procedural adjusted models included age, sex, diabetes, symptom-to-balloon time, pre-PCI TIMI flow 0–1, and high thrombus burden. Pre-analytical sensitivity models additionally adjusted for sample type, sampling time relative to PCI, freeze-thaw count, and ELISA batch. Additional analyses were restricted to samples with freeze-thaw count ≤ 1 or to the 127 pre-PCI samples (**Supplementary Table S10**).

Myocardial injury models adjusted for age, sex, symptom-to-balloon time, culprit LAD or left main, pre-PCI TIMI flow 0–1, angiographic no-reflow, and eGFR. For secondary model-performance assessment, a clinical model was compared with the same model plus NET-IP using area under the receiver operating characteristic curve (AUC), Brier score, DeLong testing, likelihood-ratio testing, calibration plotting, and decision-curve analysis. Prediction-related reporting was interpreted cautiously and aligned with transparent model-reporting principles (28).

## 3 Results

### 3.1 Cohort assembly, baseline profile, and angiographic adjudication

The final analysis cohort included 742 patients with STEMI undergoing primary PCI; 632 had normal reflow and 110 developed angiographic no-reflow (**Figure 1**). NET-IP was calculable in 725 patients and unavailable in 17. Patients with no-reflow were older than those with normal reflow and had more diabetes, chronic kidney disease, and Killip class ≥ 3, whereas sex, body mass index, smoking status, hypertension, dyslipidemia, prior myocardial infarction, prior PCI, prior stroke, symptom-to-balloon time, and cardiogenic shock at presentation were not significantly different or were only weakly imbalanced (**Table 2**). Missingness was low for main-cohort analytic variables; NET-associated ELISA markers were not assayed outside the nested biomarker subcohort and were therefore not treated as ordinary missing values (**Supplementary Table S2**).

**Figure 1:**
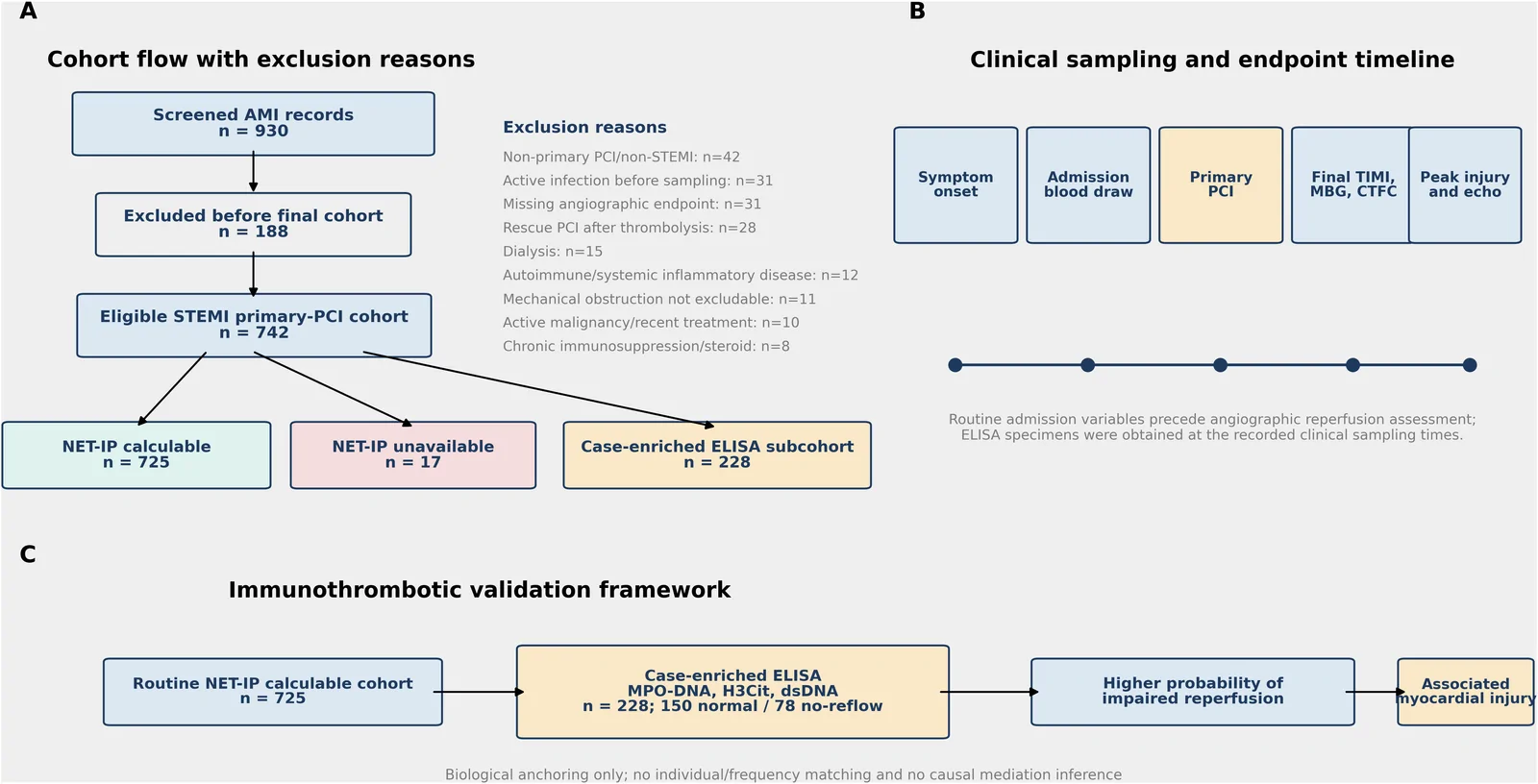
Study design, sampling timeline, and immunothrombotic validation framework. **(A)** Cohort assembly and exclusion reasons, including NET-IP calculability among the 742 eligible STEMI patients. **(B)** Clinical sampling and endpoint timeline. **(C)** Relationship between the routine NET-IP phenotype and the case-enriched nested ELISA biomarker subcohort. The ELISA subcohort comprised 150 normal-reflow and 78 no-reflow patients and was not individually or frequency matched. The framework illustrates biological anchoring rather than causal mediation.

**Table 2:** Baseline characteristics stratified by angiographic no-reflow.

Characteristic	Overall (*n* = 742)	Normal reflow (*n* = 632)	No-reflow (*n* = 110)	*P* value
Age, years	62.0 [56.0, 70.0]	61.0 [55.0, 69.0]	67.0 [60.0, 72.0]	< 0.001
Male sex	540 (72.8%)	452 (71.5%)	88 (80.0%)	0.065
Body mass index, kg/m²	24.4 [22.4, 26.4]	24.3 [22.4, 26.3]	24.9 [22.5, 26.9]	0.219
Current smoking	479 (64.6%)	411 (65.0%)	68 (61.8%)	0.516
Hypertension	413 (55.7%)	354 (56.0%)	59 (53.6%)	0.643
Diabetes mellitus	247 (33.3%)	187 (29.6%)	60 (54.5%)	< 0.001
Dyslipidemia	370 (49.9%)	315 (49.8%)	55 (50.0%)	0.976
Chronic kidney disease	75 (10.1%)	53 (8.4%)	22 (20.0%)	< 0.001
Prior myocardial infarction	83 (11.2%)	74 (11.7%)	9 (8.2%)	0.279
Prior PCI	85 (11.5%)	74 (11.7%)	11 (10.0%)	0.604
Prior stroke	76 (10.2%)	67 (10.6%)	9 (8.2%)	0.440
Symptom-to-balloon time, h	3.84 [2.86, 5.10]	3.82 [2.82, 5.07]	4.05 [3.26, 5.18]	0.172
Killip class	—	—	—	< 0.001
1	544 (73.3%)	485 (76.7%)	59 (53.6%)	
2	144 (19.4%)	115 (18.2%)	29 (26.4%)	
3	49 (6.6%)	29 (4.6%)	20 (18.2%)	
4	5 (0.7%)	3 (0.5%)	2 (1.8%)	
Killip class ≥ 3	54 (7.3%)	32 (5.1%)	22 (20.0%)	< 0.001
Cardiogenic shock at presentation	37 (5.0%)	32 (5.1%)	5 (4.5%)	0.818

Values are median [IQR] or n (%). P values are from Wilcoxon rank-sum, chi-square, or Fisher exact tests as appropriate. PCI denotes percutaneous coronary intervention.

Angiographic adjudication showed strong agreement for the primary no-reflow classification (Cohen kappa, 0.866; 95% CI, 0.812–0.917) and high agreement for MBG and CTFC (**Supplementary Table S14**). Final adjudicated MBG and CTFC were available for all 742 patients (**Table 1**). Mechanical epicardial obstruction was excluded by blinded adjudication, and residual stenosis > 30% was not present in the analysis dataset (**Table 1**).

### 3.2 Admission immunothrombotic profile and NET-IP construction

Compared with patients with normal reflow, those with no-reflow had higher admission white blood cell, neutrophil, platelet, NLR, SII, platelet-neutrophil product, hsCRP, D-dimer, fibrinogen, admission glucose, and creatinine levels, together with lower lymphocyte count and eGFR (**Table 3**). The NET-IP score was higher in no-reflow patients than in normal-reflow patients (0.73 [0.24, 1.25] vs. -0.06 [-0.77, 0.54]; *P* < 0.001), and the high NET-IP phenotype was more frequent in the no-reflow group (63.0% vs. 28.2%; *P* < 0.001) (**Table 3**).

**Table 3:** Admission laboratory and NET-associated immunothrombotic profile.

Characteristic	Overall (*n* = 742)	Normal reflow (*n* = 632)	No-reflow (*n* = 110)	*P* value
White blood cells, 10^9^/L	9.82 [8.49, 11.29]	9.67 [8.35, 11.09]	10.53 [9.34, 12.39]	< 0.001
Neutrophils, 10^9^/L	6.51 [5.07, 8.08]	6.30 [4.91, 7.84]	7.61 [6.06, 9.07]	< 0.001
Lymphocytes, 10^9^/L	1.34 [1.08, 1.63]	1.37 [1.10, 1.67]	1.17 [0.96, 1.47]	< 0.001
Platelets, 10^9^/L	225 [194, 258]	221 [193, 256]	236 [212, 269]	< 0.001
Neutrophil-to-lymphocyte ratio	4.73 [3.34, 6.81]	4.54 [3.18, 6.51]	6.19 [4.25, 8.84]	< 0.001
Systemic immune-inflammation index	1042 [716, 1560]	997 [675, 1471]	1443 [1024, 2158]	< 0.001
Platelet-neutrophil product	1423.6 [1063.4, 1911.1]	1388.8 [1023.1, 1830.8]	1790.8 [1403.2, 2299.6]	< 0.001
hsCRP, mg/L	5.94 [3.67, 9.62]	5.58 [3.48, 9.06]	8.10 [5.78, 13.91]	< 0.001
D-dimer, mg/L FEU	0.55 [0.32, 0.96]	0.50 [0.28, 0.90]	0.87 [0.56, 1.44]	< 0.001
Fibrinogen, g/L	3.12 [2.66, 3.67]	3.04 [2.62, 3.56]	3.51 [2.93, 4.27]	< 0.001
Admission glucose, mmol/L	7.53 [6.35, 9.01]	7.47 [6.32, 8.95]	8.16 [6.50, 9.44]	0.017
Creatinine, µmol/L	83.1 [72.5, 95.4]	82.5 [71.4, 94.4]	87.0 [78.3, 100.5]	0.001
eGFR, ml/min/1.73 m²	94.3 [83.6, 104.8]	95.2 [84.7, 105.2]	89.7 [76.2, 99.9]	< 0.001
NET-IP score	0.04 [-0.70, 0.66]	-0.06 [-0.77, 0.54]	0.73 [0.24, 1.25]	< 0.001
High NET-IP phenotype	242 (33.4%)	174 (28.2%)	68 (63.0%)	< 0.001

Values are median [IQR] or n (%). NET-IP denotes neutrophil extracellular trap-associated immunothrombotic phenotype; eGFR, estimated glomerular filtration rate; hsCRP, high-sensitivity C-reactive protein.

Across NET-IP tertiles, the distributions of neutrophil count, platelet-neutrophil product, hsCRP, D-dimer, fibrinogen, NLR, and SII differed across categories, whereas eGFR did not differ materially; global comparisons are summarized in **Supplementary Table S3**. Patient-level component visualization showed enrichment of the component signals across the NET-IP distribution (**Figure 2A**).

**Figure 2:**
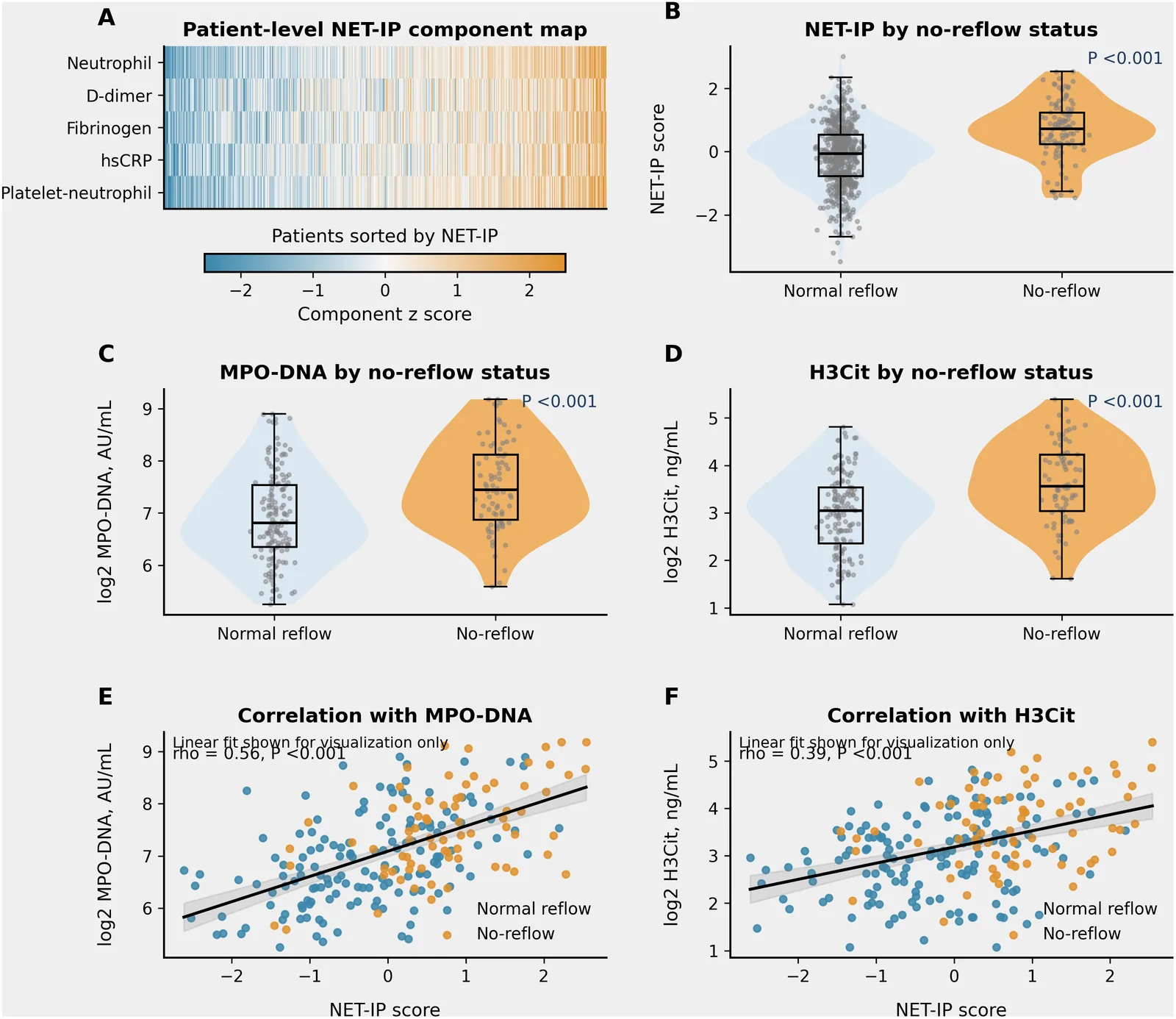
NET-IP phenotype construction and case-enriched nested ELISA biomarker validation. **(A)** Patient-level visualization of NET-IP component z scores among patients with a calculable score. **(B)** NET-IP distribution by angiographic no-reflow status. **(C, D)** Log_2_-transformed MPO-DNA and H3Cit levels by no-reflow status in the case-enriched nested ELISA biomarker subcohort. **(E, F)** Correlations between NET-IP and MPO-DNA or H3Cit; linear fitted lines are shown for visualization only, whereas correlations were assessed using Spearman rank correlation. Group sizes and P values are displayed within the relevant panels.

### 3.3 Nested ELISA biomarker validation of the NET-associated phenotype

The nested ELISA biomarker subcohort included 228 patients (150 with normal reflow and 78 with no-reflow) and was case-enriched for no-reflow without individual or frequency matching. Compared with the non-ELISA cohort, the ELISA subcohort had a deliberately higher proportion of no-reflow cases, while other baseline and procedural characteristics are summarized separately (**Supplementary Table S4**). Clinical characteristics within the ELISA subcohort are compared directly between normal-reflow and no-reflow groups in **Supplementary Table S5**. Of the 228 samples, 127 (55.7%) were obtained before PCI and 101 (44.3%) after PCI; post-PCI sampling comprised 53 immediate post-PCI samples, 25 at 6h, and 23 at 24h (**Supplementary Table S6**).

MPO-DNA, H3Cit, and dsDNA concentrations were higher in no-reflow patients than in normal-reflow patients in the ELISA subcohort (**Supplementary Table S7**; **Figure 2C** and **2D**). NET-IP correlated with measured NET-associated markers, with Spearman rho values of 0.557 for MPO-DNA, 0.388 for H3Cit, and 0.506 for dsDNA (all *P* < 0.001; **Supplementary Table S8**; **Figures 2E, F**). In clinical/procedural adjusted models, MPO-DNA (OR per SD log_2_, 1.88; 95% CI, 1.26–2.81; *P* = 0.002) and H3Cit (OR per SD log_2_, 2.01; 95% CI, 1.33–3.02; *P* < 0.001) remained associated with no-reflow, whereas dsDNA was attenuated (OR per SD log_2_, 1.41; 95% CI, 0.97–2.07; *P* = 0.074) (**Table 4**; **Supplementary Table S9**). Additional pre-analytical adjustment supported MPO-DNA and H3Cit. In the pre-PCI-only analysis, H3Cit remained associated with no-reflow, whereas the MPO-DNA estimate was attenuated (OR 1.74; 95% CI, 0.98–3.08; *P* = 0.059) (**Supplementary Table S10**).

**Table 4:** NET-IP, ELISA markers, and myocardial injury outcomes.

Domain	Outcome	Effect (95% CI)	P value
Case-enriched nested ELISA validation	Adjusted OR per SD log_2_ MPO-DNA for no-reflow	1.88 (1.26–2.81)	0.002
	Adjusted OR per SD log_2_ H3Cit for no-reflow	2.01 (1.33–3.02)	< 0.001
	Adjusted OR per SD log_2_ dsDNA for no-reflow	1.41 (0.97–2.07)	0.074
Myocardial injury	Peak hs-cTnI, percent difference per SD NET-IP	50.10 (42.47–58.13)	< 0.001
	Peak CK-MB, percent difference per SD NET-IP	29.52 (24.17–35.10)	< 0.001
	LVEF, absolute percentage-point difference per SD NET-IP	-1.08 (-1.48 to -0.68)	< 0.001
	Large myocardial injury, OR per SD NET-IP	2.70 (2.10–3.49)	< 0.001
	Discharge LVEF < 50%, OR per SD NET-IP	1.37 (1.12–1.67)	0.002

ELISA marker associations are adjusted ORs per SD log_2_ marker in the case-enriched nested biomarker subcohort, adjusted for age, sex, diabetes, symptom-to-balloon time, pre-PCI TIMI 0–1, and high thrombus burden. Additional pre-analytical adjustment and freeze-thaw sensitivity analyses are provided in **Supplementary** **Supplementary Table S8**. MPO-DNA and H3Cit are the confirmatory ELISA markers; dsDNA is supportive and less robust. Myocardial injury models adjusted for age, sex, symptom-to-balloon time, culprit LAD/left main, pre-PCI TIMI 0–1, angiographic no-reflow, and eGFR. Continuous myocardial injury outcomes were log-transformed; effects for hs-cTnI and CK-MB are percent differences per SD increase in NET-IP score.

### 3.4 NET-IP and coronary reperfusion failure

No-reflow incidence differed across NET-IP tertiles: 14 of 242 patients in the low tertile, 26 of 241 in the intermediate tertile, and 68 of 242 in the high tertile (global *P* < 0.001; **Figure 3A**). Adjusted restricted cubic spline visualization showed a positive dose-response pattern between NET-IP and no-reflow risk (**Figure 3B**). Adjudicated CTFC also differed across tertiles, with median [IQR] values of 21.1 [16.9, 24.8], 22.2 [18.9, 27.6], and 24.8 [20.5, 34.0] (Kruskal–Wallis *P* < 0.001; **Figure 3C**). MBG distribution likewise differed across NET-IP categories (global *P* < 0.001; **Figure 3D**).

**Figure 3:**
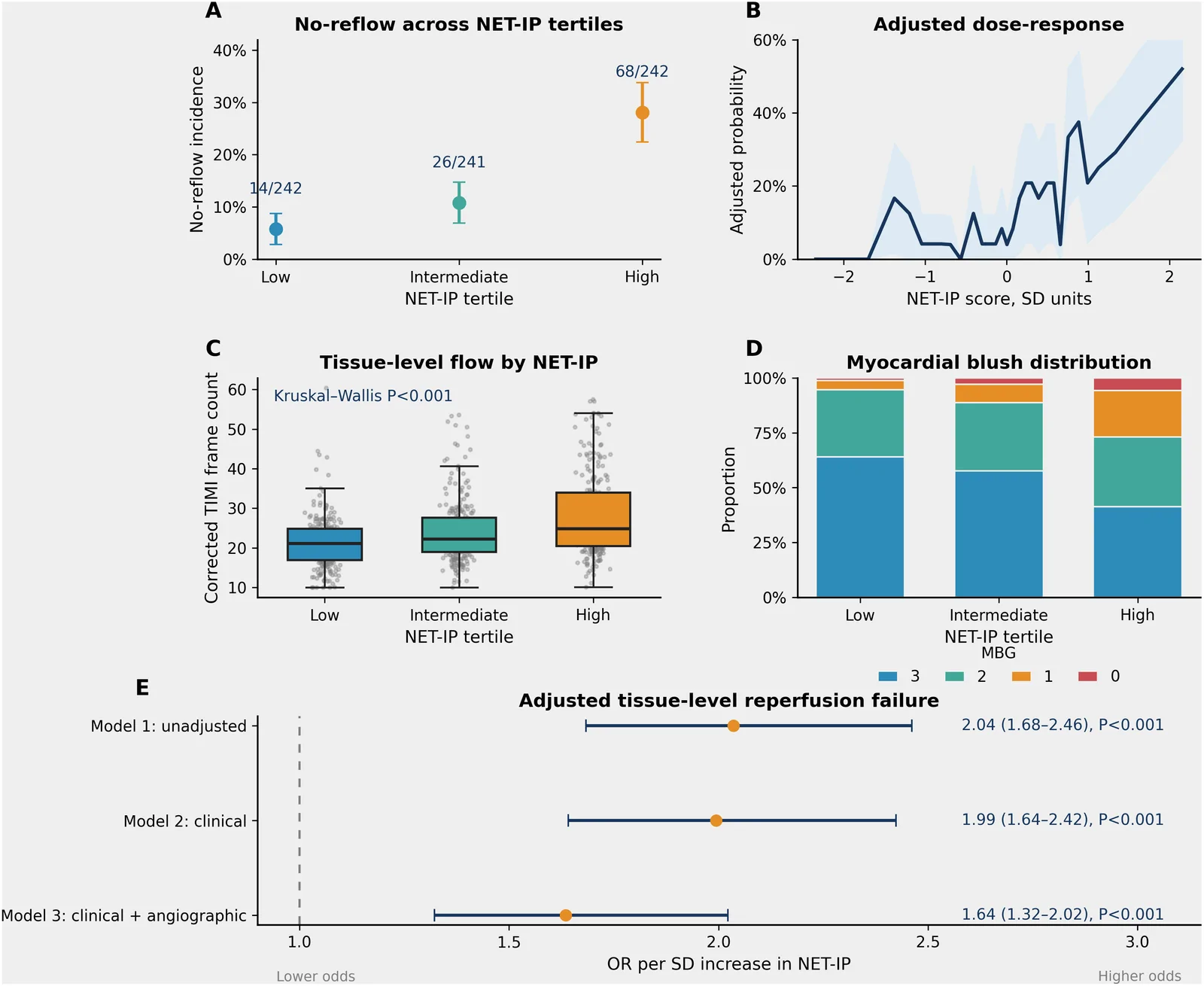
NET-IP score and coronary reperfusion failure. **(A)** No-reflow incidence across NET-IP tertiles (*n* = 242, 241, and 242; global chi-square *P* < 0.001). **(B)** Adjusted restricted cubic spline visualization of no-reflow probability across the NET-IP distribution. **(C)** Adjudicated corrected TIMI frame count across NET-IP tertiles; medians [IQR] were 21.1 [16.9, 24.8], 22.2 [18.9, 27.6], and 24.8 [20.5, 34.0] (Kruskal–Wallis *P* < 0.001). **(D)** Adjudicated myocardial blush grade distribution across NET-IP tertiles. **(E)** Sequential logistic models for tissue-level reperfusion failure, defined as final TIMI flow < 3 or final TIMI 3 with MBG 0–1 or adjudicated CTFC > 27; ORs are per SD increase in NET-IP.

In sequential logistic regression models, NET-IP was associated with angiographic no-reflow in the unadjusted model (OR per SD, 2.58; 95% CI, 2.00–3.33; *P* < 0.001), the clinical model (OR per SD, 2.50; 95% CI, 1.91–3.27; *P* < 0.001), and the clinical plus angiographic model (OR per SD, 1.96; 95% CI, 1.46–2.62; *P* < 0.001) (**Table 5**; **Figure 4A**). With tissue-level reperfusion failure defined using adjudicated CTFC > 27, 221 events occurred overall and 210 events occurred in the 700-patient complete-case set; the fully adjusted association was OR 1.64 per SD (95% CI, 1.32–2.02; *P* < 0.001) (**Table 5**; **Figure 3E**).

**Table 5:** Sequential adjusted associations between NET-IP and reperfusion failure.

Outcome	Model	N/events	OR (95% CI)	*P* value	AUC	Brier score
Angiographic no-reflow	Model 1: unadjusted	700/103	2.58 (2.00–3.33)	< 0.001	0.734	0.112
	Model 2: clinical	700/103	2.50 (1.91–3.27)	< 0.001	0.777	0.106
	Model 3: clinical + angiographic	700/103	1.96 (1.46–2.62)	< 0.001	0.817	0.100
Tissue-level reperfusion failure	Model 1: unadjusted	700/210	2.04 (1.68–2.46)	< 0.001	0.678	0.192
	Model 2: clinical	700/210	1.99 (1.64–2.42)	< 0.001	0.705	0.185
	Model 3: clinical + angiographic	700/210	1.64 (1.32–2.02)	< 0.001	0.757	0.171

Model 2 adjusted for age, sex, diabetes, and symptom-to-balloon time. Model 3 additionally adjusted for Killip class ≥ 3, culprit LAD/left main, pre-PCI TIMI 0–1, high thrombus burden, admission glucose, and eGFR. ORs are per SD increase in NET-IP score.

**Figure 4:**
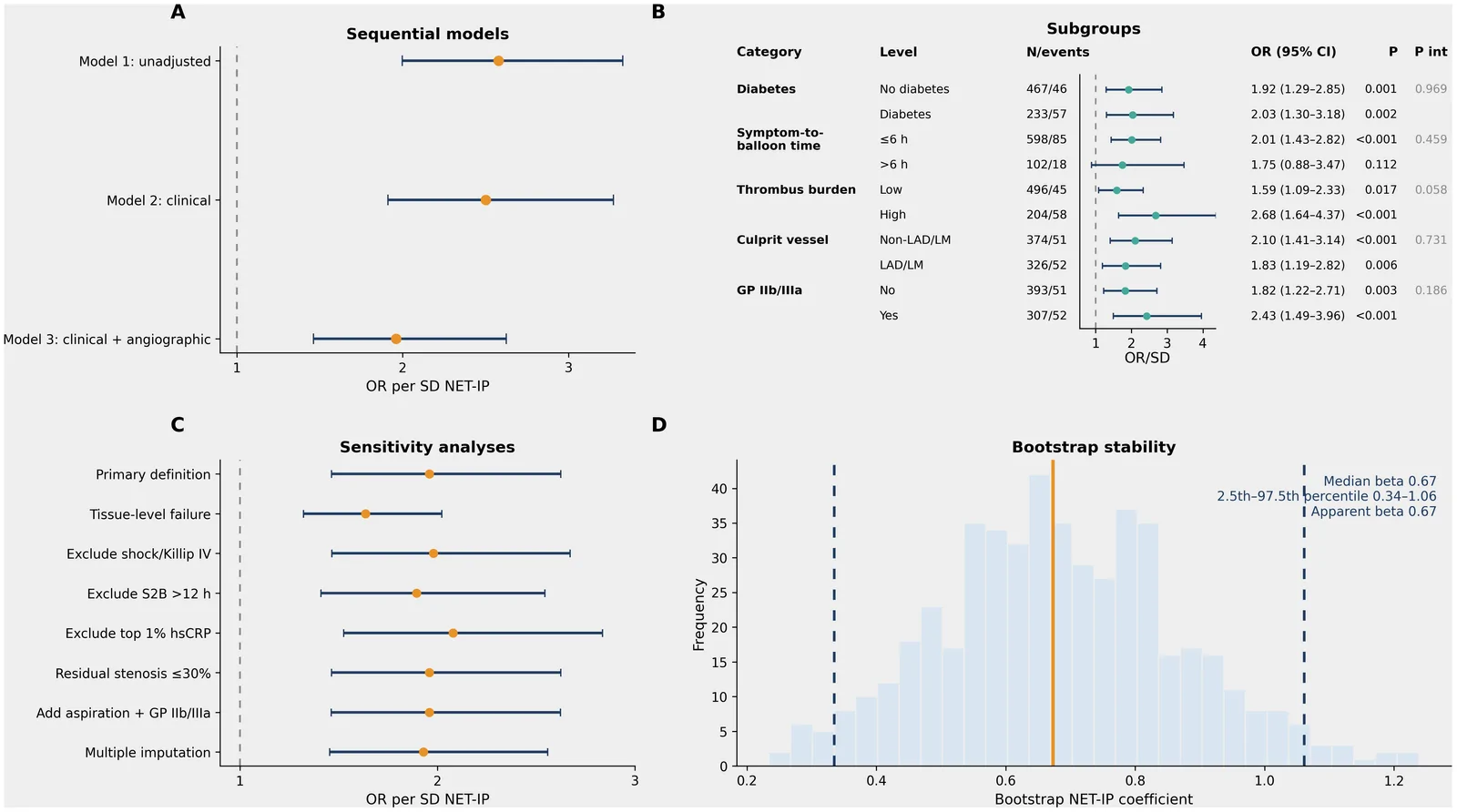
Adjusted associations, subgroup analyses, sensitivity analyses, and bootstrap stability. **(A)** Sequential logistic models for angiographic no-reflow; ORs are per SD increase in NET-IP. **(B)** Table-style subgroup forest plot showing subgroup category/level, N/events, adjusted OR per SD with 95% CI, subgroup-level P value, and P for interaction from exposure-by-subgroup interaction terms. **(C)** Sensitivity analyses, including the corrected tissue-level endpoint and multiple imputations. **(D)** Bootstrap distribution of the NET-IP coefficient in the fully adjusted no-reflow model. No subgroup interaction met the conventional *P* < 0.05 threshold.

### 3.5 Robustness, subgroup analyses, and internal validation

The main association was consistent across sensitivity analyses, including the corrected tissue-level reperfusion endpoint, exclusion of shock or Killip IV, exclusion of symptom-to-balloon time > 12 h, exclusion of the top 1% of hsCRP values, restriction to residual stenosis ≤ 30%, additional adjustment for thrombus aspiration and glycoprotein (GP) IIb/IIIa inhibitor use, and multiple imputation restricted to patients with a calculable NET-IP score (**Supplementary Table S11**; **Figure 4C**). Alternative NET-IP constructions that removed neutrophil count while retaining platelet-neutrophil product, replaced platelet-neutrophil product with platelet count, replaced neutrophil count with NLR, or used domain-balanced weighting yielded materially similar estimates (**Supplementary Table S11**).

Subgroup analyses showed directionally consistent associations across diabetes status, symptom-to-balloon time, thrombus burden, culprit vessel category, and GP IIb/IIIa inhibitor use (**Supplementary Table S12**; **Figure 4B**). No interaction reached conventional statistical significance; the thrombus-burden interaction *P* value was 0.058. Bootstrap validation of the fully adjusted model yielded an apparent AUC of 0.817, mean optimism of 0.021, optimism-corrected AUC of 0.796, and a stable bootstrap distribution of the NET-IP coefficient (**Supplementary Table S15**; **Figure 4D**).

### 3.6 NET-IP, myocardial injury, and secondary model performance

NET-IP was associated with greater myocardial injury and lower early systolic function (**Figures 5A–D**). Per SD increase in NET-IP, peak hs-cTnI was 50.10% higher (95% CI, 42.47–58.13; *P* < 0.001), peak CK-MB was 29.52% higher (95% CI, 24.17–35.10; *P* < 0.001), and LVEF was 1.08 percentage points lower (95% CI, -1.48 to -0.68; *P* < 0.001) (**Table 4**). NET-IP was also associated with large myocardial injury (OR per SD, 2.70; 95% CI, 2.10–3.49; *P* < 0.001) and discharge LVEF < 50% (OR per SD, 1.37; 95% CI, 1.12–1.67; *P* = 0.002) (**Table 4**). Benjamini-Hochberg-adjusted *P* values remained below 0.05 for these outcome families (**Supplementary Table S13**).

**Figure 5:**
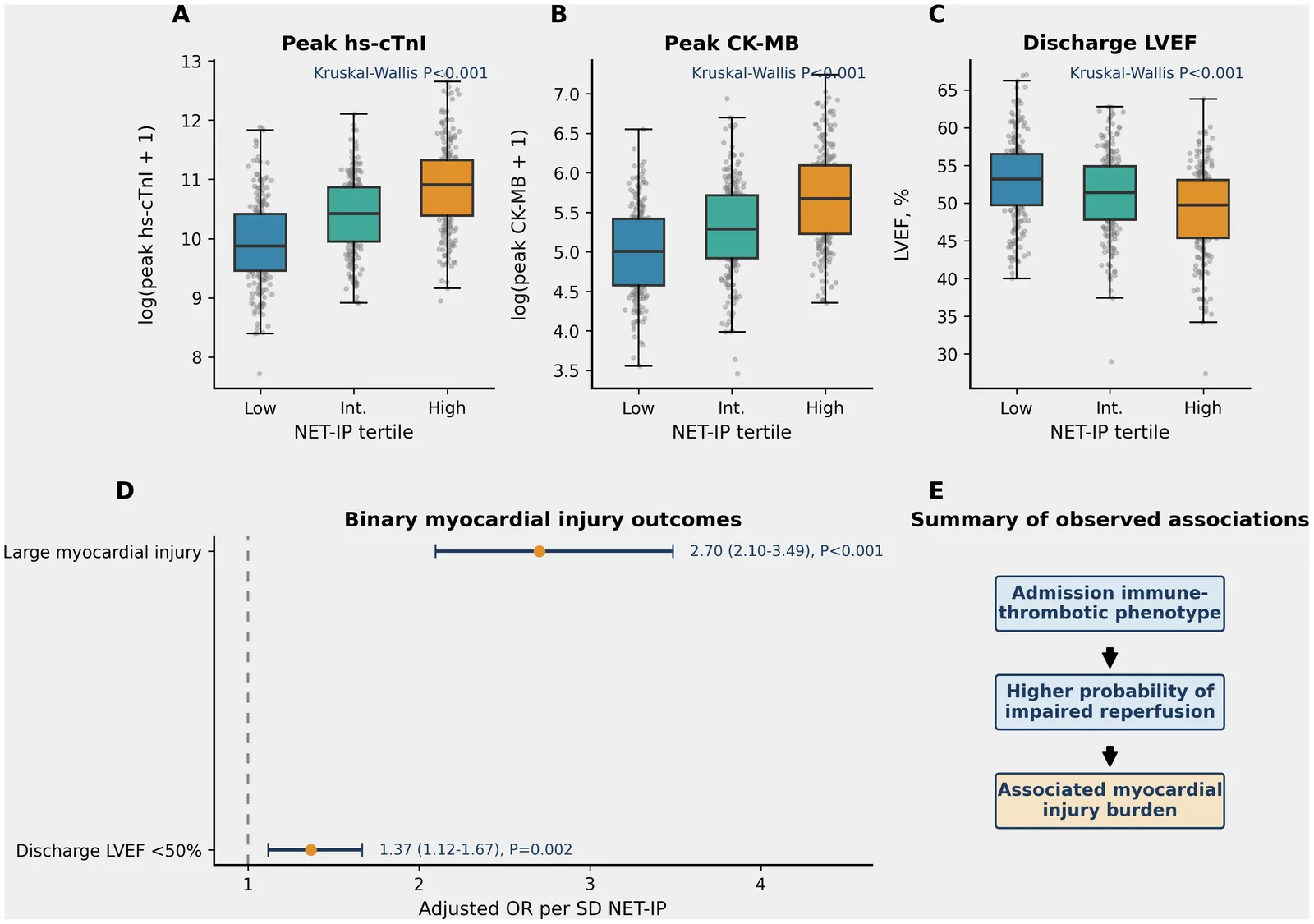
NET-IP score, myocardial injury, and summary of observed associations. **(A)** Log-transformed peak hs-cTnI, **(B)** log-transformed peak CK-MB, and **(C)** discharge LVEF across NET-IP tertiles; boxplots show median and interquartile range with individual observations, and Kruskal–Wallis *P* values are displayed in each panel. **(D)** Adjusted ORs per SD NET-IP for large myocardial injury and discharge LVEF < 50%, with 95% CIs and *P* values. **(E)** Conceptual summary of the observed associations; this panel does not represent causal mediation.

Adding NET-IP to the clinical model modestly improved secondary model-performance metrics: AUC increased from 0.793 to 0.817, and Brier score improved from 0.106 to 0.100 (**Supplementary Tables S16**-**S17**; **Supplementary Figure S5**). These analyses were considered supportive and were not used to frame the study as a clinical prediction model.

## 4 Discussion

In this real-world translational cohort of patients with STEMI undergoing primary PCI, a defined NET-associated immunothrombotic phenotype was associated with angiographic no-reflow, tissue-level reperfusion failure, and myocardial injury. The association persisted after adjustment for age, sex, diabetes, symptom-to-balloon time, Killip class, culprit vessel, pre-PCI TIMI flow, high thrombus burden, admission glucose, and renal function. The biomarker analyses should be interpreted as biological anchoring rather than direct quantification of NET burden: NET-IP correlated with MPO-DNA and H3Cit, but pre-PCI-only sensitivity attenuated the MPO-DNA estimate while preserving the H3Cit association.

The findings are coherent with the current view of coronary no-reflow as a microvascular reperfusion disorder rather than a simple failure of epicardial PCI. Reviews of no-reflow emphasize that distal embolization, microvascular plugging, endothelial injury, inflammatory vasoconstriction, and reperfusion injury often act together (1, 2). The present study adds a human clinical layer to that framework by linking no-reflow to a phenotype that integrates neutrophil activation, fibrin turnover, fibrinogen-rich acute-phase response, systemic inflammation, and platelet-neutrophil interaction. It is notable that the association remained after accounting for pre-PCI TIMI 0–1 flow and high thrombus burden, suggesting that NET-IP is not simply a surrogate for angiographic thrombus severity.

This interpretation also fits a broader thromboinflammatory framework in which platelet activation, coagulation, innate immune signaling, and neutrophil responses reinforce one another after STEMI. Recent clinical data link thromboinflammation with adverse outcome after STEMI, while experimental and clinical work indicates that antithrombotic pharmacology can modify post-ischemic thromboinflammatory signaling (29, 30). These observations provide biological context for the present associations but do not establish a treatment effect of any medication in this cohort.

The connection between NETs and coronary reperfusion failure is biologically plausible. NETs were first identified as extracellular chromatin structures that immobilize pathogens, but subsequent vascular studies showed that extracellular DNA traps can provide a scaffold for platelet adhesion and thrombus propagation (14, 15). Immunothrombosis describes the same convergence of innate immunity and coagulation inside vessels, particularly in microvascular beds (16). In AMI, such a process could be harmful if chromatin, neutrophil granule proteins, platelets, and fibrin accumulate in the downstream microcirculation after recanalization.

Experimental evidence supports this interpretation. In a myocardial ischemia-reperfusion model, NETs were detected in reperfused myocardium and were linked to microthrombosis and no-reflow; DNase-based therapy reduced NET accumulation and improved perfusion in that preclinical context (23). NET-fibrin interactions can stabilize thrombi and modify clot structure, giving a mechanistic basis for impaired washout or fibrinolysis in small vessels (17, 31). These studies do not prove that NETs caused no-reflow in our cohort, but they make the observed clinical associations mechanistically credible.

Human coronary thrombus studies also point in the same direction. Mangold and colleagues found that NET burden in culprit-site thrombi from ST-elevation acute coronary syndrome patients was related to ST-segment resolution and infarct size (19). Hofbauer and colleagues reported higher culprit-site NET surrogate markers than peripheral markers in STEMI and linked them to infarct size and ventricular dysfunction (32). Our study differs because it focused on a routinely measurable admission phenotype and angiographic no-reflow, but the ELISA results for MPO-DNA and H3Cit provide a bridge to those direct biomarker studies.

The results also align with circulating NET-marker studies after STEMI, although our interpretation of dsDNA is deliberately conservative. Helseth and colleagues reported that dsDNA levels after STEMI were associated with infarct size, left ventricular remodeling, and outcomes (21). Langseth and colleagues found associations between NET-related components, clinical outcome, and hypercoagulability after STEMI (22). In our cohort, dsDNA was higher in no-reflow patients and correlated with NET-IP, but its adjusted association was weaker and less robust than MPO-DNA and H3Cit. This pattern is biologically reasonable because dsDNA can originate from several forms of cell injury and is more vulnerable to non-NET–specific interpretation.

The comparison with conventional inflammatory indices is important. NLR and SII have been studied in STEMI and no-reflow, and they are attractive because they are easy to calculate (24, 25). However, those indices primarily summarize leukocyte and platelet counts and do not directly incorporate fibrin turnover, fibrinogen, or systemic acute-phase biology. In the current sensitivity analyses, replacing NET-IP with neutrophil count, NLR, or SII produced smaller effect estimates. This does not prove superiority in a formal biomarker-discovery sense, but it supports the idea that a structured immunothrombotic phenotype contains information beyond generic inflammation.

A separate structural concern is that neutrophil count contributes both directly and through the platelet-neutrophil product. The association was therefore re-estimated using alternative constructions that removed the direct neutrophil term, replaced the platelet-neutrophil product with platelet count, replaced neutrophil count with NLR, or balanced the cellular and noncellular domains. The fully adjusted ORs remained approximately 2.0 per SD across these alternatives (**Supplementary Table S11**), indicating that the main association was not dependent on duplicating the neutrophil contribution.

The myocardial injury findings help to connect the reperfusion endpoint with clinically meaningful myocardial damage. NET-IP was associated with higher peak hs-cTnI and CK-MB, lower discharge LVEF, large myocardial injury, and LVEF < 50%. These associations are consistent with literature showing that microvascular obstruction and impaired myocardial reperfusion are linked to adverse ventricular remodeling and outcomes after STEMI (3, 7). We did not perform causal mediation, and no-reflow should not be presented as the sole pathway from NET-IP to myocardial injury. A more cautious interpretation is that NET-associated immunothrombosis marks a systemic and coronary milieu in which both microvascular reperfusion failure and myocardial injury are more likely.

The modest improvement in model performance deserves careful framing. Adding NET-IP increased AUC from 0.793 to 0.817 and improved the Brier score from 0.106 to 0.100. These changes support incremental information, but they are not large enough to justify a clinical decision tool without external validation. This is why the manuscript is better positioned as translational immunology rather than prediction-model development. Transparent reporting principles for prediction models are still useful for describing model performance, calibration, and optimism, but the biological question remains primary (28).

An established example is the PIANO score, which combines age, absence of pre-infarction angina, total ischemic time, LAD culprit artery, pre-PCI TIMI flow, and thrombus grade to stratify angiographic no-reflow risk in patients with STEMI undergoing primary PCI (33). The present dataset contained most of these domains but did not capture pre-infarction angina with sufficient reliability; therefore, an exact head-to-head reconstruction of the PIANO score would have been methodologically incomplete. NET-IP should be viewed as a complementary immunothrombotic phenotype rather than a replacement for established clinical risk scores, and external head-to-head comparison remains necessary.

One implication is that NET-associated immunothrombosis could help define enrichment strategies for future reperfusion-injury studies. Cardiovascular inflammation trials have shown that immune pathways can be targeted in atherosclerotic disease, but the acute AMI setting is more complex because inflammation has both injurious and reparative roles (13, 34). The current phenotype may help identify patients with a high immunothrombotic burden in whom anti-NET, thromboinflammatory, or microvascular-protective strategies could be tested. Such use remains hypothetical; this observational study does not support immediate treatment selection.

The study has several strengths. The main cohort had more than 100 no-reflow events, allowing clinically selected multivariable adjustment. The angiographic endpoint was adjudicated with high interobserver agreement, and mechanical obstruction was actively excluded. The primary routine-data phenotype was defined before the present robustness analyses, while the nested biomarker subcohort provided biological support using MPO-DNA and H3Cit. Robustness analyses addressed the corrected tissue-level reperfusion endpoint, severe and delayed presentation, hsCRP extremes, procedural factors, alternative NET-IP constructions, multiple imputation, bootstrap stability, and pre-analytical ELISA factors.

Several limitations must be emphasized. First, the study was single-center and observational, so residual confounding and center-specific practice effects cannot be excluded. Second, NET-IP is an indirect phenotype and should not be interpreted as a direct measurement of NET burden. Third, the ELISA subcohort was deliberately enriched for no-reflow and was not matched, so biomarker prevalence and effect sizes should not be extrapolated to an unselected STEMI population. Fourth, only 127 of 228 ELISA samples (55.7%) were obtained before PCI; 101 (44.3%) were collected after PCI, and the pre-PCI-only analysis attenuated the MPO-DNA association. Fifth, procedural and pharmacological factors could not be exhaustively controlled despite additional sensitivity analyses. Sixth, cardiac magnetic resonance was not available to quantify microvascular obstruction or infarct size. Finally, the study lacks external validation, and the observed incremental model-performance gain is insufficient to support clinical implementation.

In summary, a clinically measurable NET-associated immunothrombotic phenotype was associated with coronary no-reflow, tissue-level reperfusion failure, and myocardial injury after primary PCI. MPO-DNA and H3Cit provided biological support for the NET-associated interpretation in a case-enriched nested biomarker subcohort, but the phenotype should not be considered a direct measure of NET burden. The findings support further external validation and mechanistic investigation of immunothrombotic pathways in STEMI reperfusion failure.

## 5 Conclusion

A clinically measurable NET-associated immunothrombotic phenotype was associated with coronary no-reflow, tissue-level reperfusion failure, and myocardial injury after primary PCI in STEMI. MPO-DNA and H3Cit provided supportive biological anchoring in a case-enriched nested biomarker subcohort. External validation and mechanistic studies are required before clinical implementation or treatment implications can be considered.

## Data Availability

The original contributions presented in the study are included in the article/**Supplementary Material**. Further inquiries can be directed to the corresponding author.
