# Supplementary material for: A NET-associated immunothrombotic phenotype is associated with coronary no-reflow and myocardial injury after primary PCI in ST-segment elevation myocardial infarction: a real-world translational cohort
Source: Front Immunol. 2026 Sep 29;17:1926100. doi: 10.3389/fimmu.2026.1926100 (PMC13657948; doi:10.3389/fimmu.2026.1926100)
Supplement: Supplementary file 1 [file Table1.docx]

Supplementary Materials

Supplementary Tables

Supplementary Table S1. Operational variable definitions.

| **Variable** | **Definition** |
| --- | --- |
| NET-IP score | Mean of available component z scores after log transformation and component standardization for neutrophil count, D-dimer, fibrinogen, hsCRP, and platelet-neutrophil product; at least four of five components required, followed by standardization of the composite score. |
| High NET-IP phenotype | Upper tertile of the NET-IP score. |
| Angiographic no-reflow | Final TIMI flow grade <3 after PCI in the absence of mechanical epicardial obstruction. |
| Tissue-level reperfusion failure | Final TIMI flow <3 or final TIMI 3 with MBG 0-1 or adjudicated CTFC >27. |
| High thrombus burden | Pre-PCI thrombus grade consistent with high angiographic thrombus burden. |
| Large myocardial injury | Top quartile of the peak myocardial injury marker in the analysis cohort. |
| ELISA subcohort | Case-enriched nested biomarker subcohort with available residual clinical samples; 150 normal-reflow and 78 no-reflow patients; no individual or frequency matching. |
| Systemic immune-inflammation index | Platelet count × neutrophil count / lymphocyte count. |

NET-IP, neutrophil extracellular trap-associated immunothrombotic phenotype; CTFC, corrected TIMI frame count; MBG, myocardial blush grade.

Supplementary Table S2. Missing data pattern and nested ELISA not-measured status.

| **Variable** | **Missing** | **Missing percent** | **Analysis denominator** | **Interpretation** |
| --- | --- | --- | --- | --- |
| Age, years | 0 | 0.0 | Main cohort (n=742) | Ordinary missingness in the main analysis cohort. |
| Male sex | 0 | 0.0 | Main cohort (n=742) | Ordinary missingness in the main analysis cohort. |
| Body mass index, kg/m² | 0 | 0.0 | Main cohort (n=742) | Ordinary missingness in the main analysis cohort. |
| Diabetes mellitus | 0 | 0.0 | Main cohort (n=742) | Ordinary missingness in the main analysis cohort. |
| Symptom-to-balloon time, h | 0 | 0.0 | Main cohort (n=742) | Ordinary missingness in the main analysis cohort. |
| Killip class | 0 | 0.0 | Main cohort (n=742) | Ordinary missingness in the main analysis cohort. |
| Cardiogenic shock at presentation | 0 | 0.0 | Main cohort (n=742) | Ordinary missingness in the main analysis cohort. |
| Culprit vessel | 0 | 0.0 | Main cohort (n=742) | Ordinary missingness in the main analysis cohort. |
| Pre-PCI TIMI flow grade | 0 | 0.0 | Main cohort (n=742) | Ordinary missingness in the main analysis cohort. |
| Pre-PCI thrombus grade | 0 | 0.0 | Main cohort (n=742) | Ordinary missingness in the main analysis cohort. |
| High thrombus burden | 0 | 0.0 | Main cohort (n=742) | Ordinary missingness in the main analysis cohort. |
| Neutrophils, 10^9/L | 14 | 1.9 | Main cohort (n=742) | Ordinary missingness in the main analysis cohort. |
| Lymphocytes, 10^9/L | 6 | 0.8 | Main cohort (n=742) | Ordinary missingness in the main analysis cohort. |
| Platelets, 10^9/L | 3 | 0.4 | Main cohort (n=742) | Ordinary missingness in the main analysis cohort. |
| hsCRP, mg/L | 52 | 7.0 | Main cohort (n=742) | Ordinary missingness in the main analysis cohort. |
| D-dimer, mg/L FEU | 39 | 5.3 | Main cohort (n=742) | Ordinary missingness in the main analysis cohort. |
| Fibrinogen, g/L | 42 | 5.7 | Main cohort (n=742) | Ordinary missingness in the main analysis cohort. |
| Admission glucose, mmol/L | 15 | 2.0 | Main cohort (n=742) | Ordinary missingness in the main analysis cohort. |
| eGFR, mL/min/1.73 m² | 10 | 1.3 | Main cohort (n=742) | Ordinary missingness in the main analysis cohort. |
| NET-IP score | 17 | 2.3 | Main cohort (n=742) | Ordinary missingness in the main analysis cohort. |
| Angiographic no-reflow | 0 | 0.0 | Main cohort (n=742) | Ordinary missingness in the main analysis cohort. |
| Tissue-level reperfusion failure | 0 | 0.0 | Main cohort (n=742) | Ordinary missingness in the main analysis cohort. |
| Myocardial blush grade | 0 | 0.0 | Main cohort (n=742) | Final adjudicated value available for all patients. |
| Corrected TIMI frame count | 0 | 0.0 | Main cohort (n=742) | Final adjudicated value available for all patients. |
| ST-segment resolution, % | 60 | 8.1 | Main cohort (n=742) | Ordinary missingness in the main analysis cohort. |
| Peak hs-cTnI, ng/L | 28 | 3.8 | Main cohort (n=742) | Ordinary missingness in the main analysis cohort. |
| Peak CK-MB, U/L | 21 | 2.8 | Main cohort (n=742) | Ordinary missingness in the main analysis cohort. |
| Left ventricular ejection fraction, % | 34 | 4.6 | Main cohort (n=742) | Ordinary missingness in the main analysis cohort. |
| MPO-DNA, AU/mL | 514 | 69.3 | Nested ELISA subcohort only (n=228) | Not assayed outside the nested ELISA subcohort; not treated as ordinary missingness. |
| H3Cit, ng/mL | 514 | 69.3 | Nested ELISA subcohort only (n=228) | Not assayed outside the nested ELISA subcohort; not treated as ordinary missingness. |
| dsDNA, ng/mL | 514 | 69.3 | Nested ELISA subcohort only (n=228) | Not assayed outside the nested ELISA subcohort; not treated as ordinary missingness. |

MPO-DNA, H3Cit, and dsDNA were not assayed outside the case-enriched nested ELISA subcohort and were not interpreted as ordinary missing data.

Supplementary Table S3. Laboratory profile across NET-IP tertiles.

| **Characteristic** | **Overall (n=725)** | **Low (n=242)** | **Intermediate (n=241)** | **High (n=242)** | **P value** |
| --- | --- | --- | --- | --- | --- |
| White blood cells, 10^9/L | 9.82 [8.49, 11.28] | 8.13 [7.28, 8.95] | 9.76 [8.95, 10.63] | 11.78 [10.54, 13.07] | <0.001 |
| Neutrophils, 10^9/L | 6.51 [5.07, 8.08] | 4.64 [3.99, 5.57] | 6.54 [5.63, 7.20] | 8.61 [7.54, 9.87] | <0.001 |
| Lymphocytes, 10^9/L | 1.33 [1.08, 1.63] | 1.52 [1.30, 1.85] | 1.37 [1.16, 1.60] | 1.12 [0.95, 1.37] | <0.001 |
| Platelets, 10^9/L | 225 [195, 258] | 208 [166, 236] | 229 [200, 261] | 239 [210, 268] | <0.001 |
| Neutrophil-to-lymphocyte ratio | 4.74 [3.35, 6.84] | 3.02 [2.33, 3.95] | 4.69 [3.80, 5.96] | 7.55 [5.81, 9.87] | <0.001 |
| Systemic immune-inflammation index | 1042 [717, 1571] | 622 [424, 846] | 1058 [864, 1362] | 1830 [1364, 2333] | <0.001 |
| Platelet-neutrophil product | 1423.5 [1063.8, 1913.7] | 924.9 [742.0, 1179.5] | 1446.4 [1247.9, 1698.5] | 2043.1 [1710.1, 2471.5] | <0.001 |
| hsCRP, mg/L | 5.98 [3.67, 9.62] | 3.52 [2.27, 5.12] | 5.87 [4.19, 8.50] | 10.11 [7.29, 15.37] | <0.001 |
| D-dimer, mg/L FEU | 0.55 [0.31, 0.96] | 0.28 [0.19, 0.45] | 0.55 [0.36, 0.87] | 1.01 [0.63, 1.46] | <0.001 |
| Fibrinogen, g/L | 3.12 [2.66, 3.70] | 2.56 [2.27, 2.97] | 3.13 [2.84, 3.49] | 3.76 [3.23, 4.22] | <0.001 |
| Admission glucose, mmol/L | 7.52 [6.35, 9.01] | 7.28 [6.29, 8.66] | 7.71 [6.46, 9.03] | 7.67 [6.37, 9.13] | 0.053 |
| Creatinine, µmol/L | 83.0 [72.5, 95.3] | 82.6 [70.8, 93.7] | 83.7 [72.7, 98.5] | 81.8 [74.7, 94.1] | 0.423 |
| eGFR, mL/min/1.73 m² | 94.3 [83.5, 104.9] | 95.5 [85.9, 105.2] | 94.7 [83.3, 105.2] | 91.6 [81.8, 104.1] | 0.150 |
| NET-IP score | 0.04 [-0.70, 0.66] | -0.99 [-1.37, -0.70] | 0.04 [-0.13, 0.24] | 0.94 [0.66, 1.35] | <0.001 |
| High NET-IP phenotype | 242 (33.4%) | 0 (0.0%) | 0 (0.0%) | 242 (100.0%) | <0.001 |

Values are median [IQR] or n (%). Global P values are from Kruskal-Wallis or chi-square tests, as appropriate.

Supplementary Table S4. Clinical characteristics of the ELISA and non-ELISA cohorts.

| **Characteristic** | **Non-ELISA cohort (n=514)** | **ELISA subcohort (n=228)** | **P value** |
| --- | --- | --- | --- |
| Age, years | 62.00 [56.00, 69.00] | 62.00 [56.00, 71.00] | 0.161 |
| BMI, kg/m² | 24.25 [22.40, 26.20] | 24.80 [22.40, 26.90] | 0.229 |
| Symptom-to-balloon time, h | 3.81 [2.80, 4.97] | 4.02 [3.04, 5.32] | 0.099 |
| Admission glucose, mmol/L | 7.52 [6.33, 9.01] | 7.56 [6.39, 8.99] | 0.863 |
| eGFR, mL/min/1.73m² | 94.90 [84.70, 105.30] | 92.80 [81.60, 102.90] | 0.036 |
| NET-IP score | -0.01 [-0.67, 0.62] | 0.19 [-0.75, 0.76] | 0.186 |
| Male sex | 372/514 (72.4%) | 168/228 (73.7%) | 0.789 |
| Current smoking | 347/514 (67.5%) | 132/228 (57.9%) | 0.013 |
| Hypertension | 283/514 (55.1%) | 130/228 (57.0%) | 0.632 |
| Diabetes mellitus | 161/514 (31.3%) | 86/228 (37.7%) | 0.092 |
| Dyslipidemia | 260/514 (50.6%) | 110/228 (48.2%) | 0.578 |
| Chronic kidney disease | 46/514 (8.9%) | 29/228 (12.7%) | 0.146 |
| Prior MI | 56/514 (10.9%) | 27/228 (11.8%) | 0.706 |
| Prior PCI | 51/514 (9.9%) | 34/228 (14.9%) | 0.060 |
| Prior stroke | 52/514 (10.1%) | 24/228 (10.5%) | 0.896 |
| Killip class ≥3 | 27/514 (5.3%) | 27/228 (11.8%) | 0.002 |
| LAD/left-main culprit | 235/514 (45.7%) | 108/228 (47.4%) | 0.690 |
| Pre-PCI TIMI 0–1 | 342/514 (66.5%) | 149/228 (65.4%) | 0.801 |
| High thrombus burden | 149/514 (29.0%) | 71/228 (31.1%) | 0.601 |
| Angiographic no-reflow | 32/514 (6.2%) | 78/228 (34.2%) | <0.001 |

The ELISA subcohort was deliberately case-enriched for no-reflow and was not intended to estimate population biomarker prevalence.

Supplementary Table S5. Clinical characteristics within the ELISA subcohort by angiographic no-reflow status.

| **Characteristic** | **Normal reflow (n=150)** | **No-reflow (n=78)** | **P value** |
| --- | --- | --- | --- |
| Age, years | 61.00 [55.00, 69.75] | 67.00 [60.00, 72.75] | <0.001 |
| BMI, kg/m² | 24.60 [22.40, 26.88] | 24.95 [22.52, 27.05] | 0.469 |
| Symptom-to-balloon time, h | 3.90 [2.96, 5.36] | 4.10 [3.27, 5.18] | 0.509 |
| Admission glucose, mmol/L | 7.34 [6.28, 8.68] | 8.22 [6.51, 9.43] | 0.018 |
| eGFR, mL/min/1.73m² | 95.55 [83.57, 104.53] | 89.60 [76.20, 97.60] | 0.003 |
| NET-IP score | -0.21 [-0.96, 0.41] | 0.73 [0.27, 1.40] | <0.001 |
| Male sex | 107/150 (71.3%) | 61/78 (78.2%) | 0.342 |
| Current smoking | 86/150 (57.3%) | 46/78 (59.0%) | 0.888 |
| Hypertension | 87/150 (58.0%) | 43/78 (55.1%) | 0.778 |
| Diabetes mellitus | 43/150 (28.7%) | 43/78 (55.1%) | <0.001 |
| Dyslipidemia | 73/150 (48.7%) | 37/78 (47.4%) | 0.890 |
| Chronic kidney disease | 14/150 (9.3%) | 15/78 (19.2%) | 0.038 |
| Prior MI | 21/150 (14.0%) | 6/78 (7.7%) | 0.198 |
| Prior PCI | 23/150 (15.3%) | 11/78 (14.1%) | 0.847 |
| Prior stroke | 17/150 (11.3%) | 7/78 (9.0%) | 0.655 |
| Killip class ≥3 | 9/150 (6.0%) | 18/78 (23.1%) | <0.001 |
| LAD/left-main culprit | 60/150 (40.0%) | 48/78 (61.5%) | 0.002 |
| Pre-PCI TIMI 0–1 | 77/150 (51.3%) | 72/78 (92.3%) | <0.001 |
| High thrombus burden | 19/150 (12.7%) | 52/78 (66.7%) | <0.001 |
| Pre-admission aspirin | 29/150 (19.3%) | 12/78 (15.4%) | 0.586 |
| Pre-admission statin | 40/150 (26.7%) | 16/78 (20.5%) | 0.335 |
| Pre-admission beta-blocker | 27/150 (18.0%) | 14/78 (17.9%) | 1.000 |
| Pre-admission ACEI/ARB/ARNI | 54/150 (36.0%) | 25/78 (32.1%) | 0.660 |

The ELISA subcohort comprised 150 normal-reflow and 78 no-reflow patients. No individual or frequency matching was used.

Supplementary Table S6. ELISA sampling timing relative to PCI.

| **Timing** | **N** | **Percent** |
| --- | --- | --- |
| pre-PCI | 127 | 55.7 |
| immediate post-PCI | 53 | 23.2 |
| 6 h post-PCI | 25 | 11.0 |
| 24 h post-PCI | 23 | 10.1 |

Pre-PCI samples accounted for 127/228 (55.7%); post-PCI samples accounted for 101/228 (44.3%).

Supplementary Table S7. ELISA markers by no-reflow status.

| **Characteristic** | **Overall (n=228)** | **Normal reflow (n=150)** | **No-reflow (n=78)** | **P value** |
| --- | --- | --- | --- | --- |
| MPO-DNA, AU/mL | 133.7 [88.8, 211.9] | 112.8 [81.5, 186.6] | 175.2 [117.5, 279.4] | <0.001 |
| H3Cit, ng/mL | 9.58 [6.16, 14.96] | 8.26 [5.12, 11.63] | 11.86 [8.23, 18.77] | <0.001 |
| dsDNA, ng/mL | 594.2 [361.4, 826.0] | 531.5 [315.0, 741.1] | 722.2 [501.1, 1069.0] | <0.001 |

Values are median [IQR].

Supplementary Table S8. Spearman correlations between NET-IP and measured NET-associated markers.

| **Marker** | **N (all samples)** | **Spearman ρ (all samples)** | **P value (all samples)** | **N (pre-PCI)** | **Spearman ρ (pre-PCI)** | **P value (pre-PCI)** | **BH-FDR P (all samples)** | **BH-FDR P (pre-PCI)** |
| --- | --- | --- | --- | --- | --- | --- | --- | --- |
| MPO-DNA | 224 | 0.557 | <0.001 | 124 | 0.602 | <0.001 | <0.001 | <0.001 |
| H3Cit | 224 | 0.388 | <0.001 | 124 | 0.415 | <0.001 | <0.001 | <0.001 |
| dsDNA | 224 | 0.506 | <0.001 | 124 | 0.575 | <0.001 | <0.001 | <0.001 |

Correlations are shown for the full biomarker subcohort and the pre-PCI subset.

Supplementary Table S9. Adjusted ELISA marker logistic models for angiographic no-reflow.

| **Marker** | **N** | **Events** | **OR (95% CI)** | **P value** | **BH-FDR P** |
| --- | --- | --- | --- | --- | --- |
| MPO-DNA | 228 | 78 | 1.88 (1.26-2.81) | 0.002 | 0.003 |
| H3Cit | 228 | 78 | 2.01 (1.33-3.02) | <0.001 | 0.003 |
| dsDNA | 228 | 78 | 1.41 (0.97-2.07) | 0.074 | 0.074 |

ORs are per SD increase in the log2-transformed marker and adjusted for age, sex, diabetes, symptom-to-balloon time, pre-PCI TIMI flow 0-1, and high thrombus burden.

Supplementary Table S10. Pre-analytical and pre-PCI sensitivity analyses for ELISA marker associations with no-reflow.

| **Analysis** | **Marker** | **N** | **Events** | **OR (95% CI)** | **P value** | **BH-FDR P** |
| --- | --- | --- | --- | --- | --- | --- |
| All samples: clinical/procedural adjusted | MPO-DNA | 228 | 78 | 1.88 (1.26-2.81) | 0.002 | 0.003 |
| All samples: + pre-analytical adjusted | MPO-DNA | 228 | 78 | 2.09 (1.33-3.27) | 0.001 | 0.002 |
| Pre-PCI only: clinical/procedural + pre-analytical adjusted | MPO-DNA | 127 | 46 | 1.74 (0.98-3.08) | 0.059 | 0.059 |
| Freeze-thaw <=1 sensitivity | MPO-DNA | 218 | 75 | 1.92 (1.28-2.89) | 0.002 | 0.003 |
| All samples: clinical/procedural adjusted | H3Cit | 228 | 78 | 2.01 (1.33-3.02) | <0.001 | 0.003 |
| All samples: + pre-analytical adjusted | H3Cit | 228 | 78 | 2.22 (1.40-3.51) | <0.001 | 0.002 |
| Pre-PCI only: clinical/procedural + pre-analytical adjusted | H3Cit | 127 | 46 | 2.34 (1.25-4.36) | 0.008 | 0.023 |
| Freeze-thaw <=1 sensitivity | H3Cit | 218 | 75 | 2.08 (1.36-3.17) | <0.001 | 0.002 |
| All samples: clinical/procedural adjusted | dsDNA | 228 | 78 | 1.41 (0.97-2.07) | 0.074 | 0.074 |
| All samples: + pre-analytical adjusted | dsDNA | 228 | 78 | 1.55 (1.01-2.38) | 0.045 | 0.045 |
| Pre-PCI only: clinical/procedural + pre-analytical adjusted | dsDNA | 127 | 46 | 2.01 (1.09-3.71) | 0.026 | 0.039 |
| Freeze-thaw <=1 sensitivity | dsDNA | 218 | 75 | 1.40 (0.95-2.06) | 0.086 | 0.086 |

Pre-analytical adjustment includes sample type, sampling time relative to PCI, freeze-thaw count, and ELISA batch. BH-FDR-adjusted P values are provided within each analysis family.

Supplementary Table S11. Sensitivity analyses for the association with angiographic no-reflow.

| **Analysis** | **N** | **Events** | **OR (95% CI)** | **P value** |
| --- | --- | --- | --- | --- |
| Primary fully adjusted | 700 | 103 | 1.96 (1.46-2.62) | <0.001 |
| + pre-admission medications | 700 | 103 | 1.96 (1.46-2.64) | <0.001 |
| + aspiration and GP IIb/IIIa | 700 | 103 | 1.96 (1.46-2.62) | <0.001 |
| + lesion/stent complexity | 700 | 103 | 2.01 (1.49-2.72) | <0.001 |
| Corrected tissue-level reperfusion failure | 700 | 210 | 1.64 (1.32-2.02) | <0.001 |
| Multiple imputation among NET-IP-calculable patients | 725 | 108 | 1.93 (1.46-2.56) | <0.001 |
| Original NET-IP | 700 | 103 | 1.96 (1.46–2.62) | <0.001 |
| Remove neutrophil; retain platelet-neutrophil product | 711 | 103 | 2.09 (1.55–2.80) | <0.001 |
| Replace platelet-neutrophil product with platelet count | 711 | 103 | 2.06 (1.53–2.78) | <0.001 |
| Replace neutrophil with NLR | 699 | 103 | 2.03 (1.51–2.73) | <0.001 |
| Domain-balanced weighting | 711 | 103 | 2.06 (1.53–2.77) | <0.001 |

ORs are per SD increase in the specified NET-IP construction unless otherwise noted. The corrected tissue-level endpoint uses adjudicated CTFC >27.

Supplementary Table S12. Subgroup analyses for angiographic no-reflow.

| **Subgroup category** | **Subgroup level** | **N** | **Events** | **OR (95% CI)** | **P value** | **P for interaction** |
| --- | --- | --- | --- | --- | --- | --- |
| Diabetes | No diabetes | 467 | 46 | 1.92 (1.29–2.85) | 0.001 | 0.969 |
| Diabetes | Diabetes | 233 | 57 | 2.03 (1.30–3.18) | 0.002 |  |
| Symptom-to-balloon time | ≤6 h | 598 | 85 | 2.01 (1.43–2.82) | <0.001 | 0.459 |
| Symptom-to-balloon time | >6 h | 102 | 18 | 1.75 (0.88–3.47) | 0.112 |  |
| Thrombus burden | Low thrombus burden | 496 | 45 | 1.59 (1.09–2.33) | 0.017 | 0.058 |
| Thrombus burden | High thrombus burden | 204 | 58 | 2.68 (1.64–4.37) | <0.001 |  |
| Culprit vessel | Non-LAD/LM | 374 | 51 | 2.10 (1.41–3.14) | <0.001 | 0.731 |
| Culprit vessel | LAD/LM | 326 | 52 | 1.83 (1.19–2.82) | 0.006 |  |
| GP IIb/IIIa inhibitor | No | 393 | 51 | 1.82 (1.22–2.71) | 0.003 | 0.186 |
| GP IIb/IIIa inhibitor | Yes | 307 | 52 | 2.43 (1.49–3.96) | <0.001 |  |

Interaction P values derive from models containing the NET-IP × subgroup interaction term and are displayed once per subgroup category in Figure 4B.

Supplementary Table S13. Multiple-imputation model specification.

| **Item** | **Specification** |
| --- | --- |
| Number of imputations | 20 |
| Maximum iterations | 20 |
| Outcome handling | Angiographic no-reflow was included as the analysis outcome and was not imputed. |
| Imputed/predictor variables | Among the 725 patients with calculable NET-IP, incomplete model covariates were imputed; the NET-IP score itself was not imputed. |

Supplementary Table S14. Interobserver agreement.

| **Endpoint** | **Statistic** | **Estimate (95% CI)** | **P value** |
| --- | --- | --- | --- |
| No-reflow classification | Cohen kappa | 0.866 (0.812-0.917) | <0.001 |
| Myocardial blush grade | Weighted kappa | 0.948 (0.929-0.964) | <0.001 |
| Corrected TIMI frame count | ICC | 0.861 (0.835-0.880) | <0.001 |

ICC, intraclass correlation coefficient.

Supplementary Table S15. Internal bootstrap validation.

| **Metric** | **Value** |
| --- | --- |
| Apparent AUC | 0.817 |
| Mean optimism | 0.021 |
| Optimism-corrected AUC | 0.796 |
| Median bootstrap NET-IP beta | 0.672 |
| Bootstrap NET-IP beta 2.5th percentile | 0.335 |
| Bootstrap NET-IP beta 97.5th percentile | 1.061 |
| Median bootstrap validation calibration slope | 0.885 |

Bootstrap validation was performed for the fully adjusted angiographic no-reflow model.

Supplementary Table S16. Model performance.

| **Model** | **AUC (95% CI)** | **Brier score** |
| --- | --- | --- |
| Clinical model | 0.793 (0.748-0.837) | 0.106 |
| Clinical model + NET-IP | 0.817 (0.772-0.862) | 0.100 |

The clinical model included the clinical and angiographic covariates used in the fully adjusted model without NET-IP.

Supplementary Table S17. Incremental model performance.

| **Metric** | **Value** |
| --- | --- |
| Delta AUC | 0.024 |
| DeLong P value | 0.036 |
| Likelihood-ratio P value | <0.001 |

Incremental model-performance analyses were secondary and supportive.

Supplementary Figures

Supplementary Figure S1. Missingness map for main-cohort analytic variables. ELISA markers are summarized separately because they were assayed only in the case-enriched nested biomarker subcohort.

Supplementary Figure S2. ELISA duplicate and internal-control coefficient of variation. The plot summarizes duplicate and internal-control assay precision for the NET-associated markers measured in the nested ELISA subcohort.

Supplementary Figure S3. Calibration of the fully adjusted angiographic no-reflow model including NET-IP.

Supplementary Figure S4. NET-IP score compared with neutrophil count, NLR, and SII. Comparator analyses evaluate whether the composite phenotype differs from routine single or composite inflammatory indices.

Supplementary Figure S5. ROC and decision-curve analyses for secondary model-performance assessment. These analyses compare the clinical model with the same model plus NET-IP and are interpreted as supportive rather than as a validated clinical prediction tool.
